# Rosai–Dorfman Disease, Presenting as a Mass in the Trachea: A Case Report

**DOI:** 10.1002/rcr2.70419

**Published:** 2025-11-29

**Authors:** Taeho Youn, Boram Lee, Joungho Han, Byeong‐Ho Jeong

**Affiliations:** ^1^ Department of Internal Medicine Aerospace Medical Center Cheongju Republic of Korea; ^2^ Division of Pulmonary and Critical Care Medicine, Department of Medicine Samsung Medical Center, Sungkyunkwan University School of Medicine Seoul Republic of Korea; ^3^ Department of Pathology Samsung Medical Center, Sungkyunkwan University School of Medicine Seoul Republic of Korea

**Keywords:** bronchoscopy, Rosai–Dorfman disease, trachea

## Abstract

A 45‐year‐old woman was referred to our clinic for evaluation of a 17 mm tracheal mass detected incidentally during a routine health check‐up. The mass was removed bronchoscopically using rigid bronchoscopy for both diagnostic and therapeutic purposes. Multidisciplinary review confirmed the diagnosis of Rosai–Dorfman disease (RDD). As the patient remained asymptomatic, the residual extratracheal lesion was initially managed with observation. However, 20 months after resection, follow‐up CT revealed progression of the extratracheal component. Consequently, systemic corticosteroid therapy was initiated and continued for 9 months. The lesion showed a marked reduction in size and has remained stable for 4 years following the completion of steroid treatment. This case highlights that rare entities such as RDD can present as tracheal masses and should be considered in the differential diagnosis. Local resection followed by corticosteroid therapy can be an effective treatment approach.

## Graphical abstract

We present a rare case of Rosai–Dorfman disease (RDD) confined solely to the trachea. After initial bronchoscopic resection, systemic corticosteroid therapy was initiated due to lesion progression, successfully stabilizing the disease for 4 years.

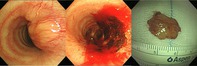

## 1 Introduction

When encountering masses in the trachea, primary tracheal tumours such as squamous cell carcinoma and adenoid cystic carcinoma are typically the first diagnostic considerations [1]. When these endotracheal lesions result in airway narrowing or distal obstruction, bronchoscopic interventions are often performed both to alleviate symptoms and to obtain adequate histologic specimens for diagnosis. We present a rare case of Rosai–Dorfman disease (RDD) confined solely to the trachea, with a focus on the diagnostic process and treatment options.

## 2 Case Report

**FIGURE 1:**
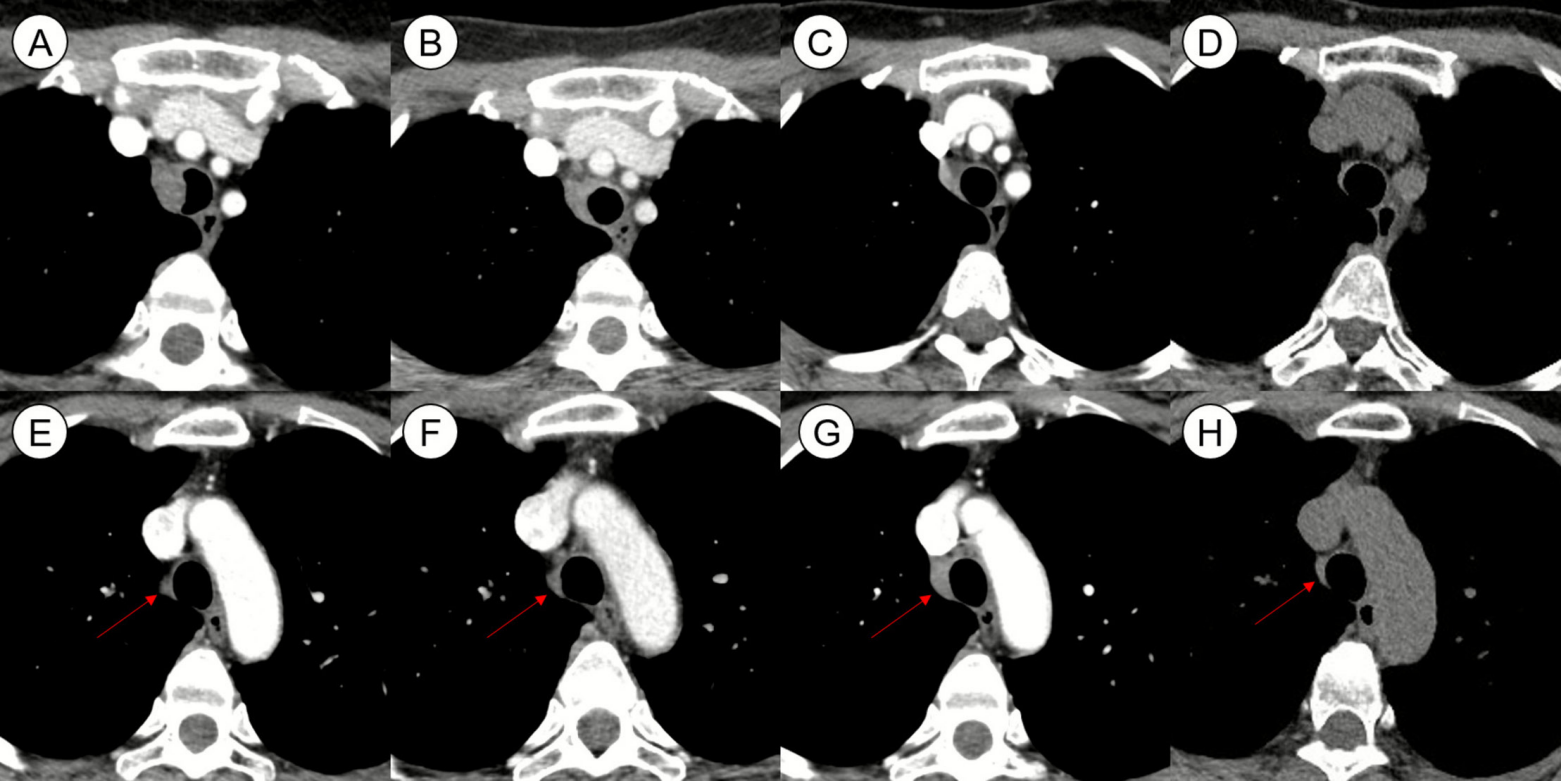
Serial chest computed tomography (CT) images of a patient with Rosai–Dorfman disease (RDD) at the T4 vertebral level. The upper row (A–D) shows axial images at the upper T4 level, and the lower row (E–H) corresponds to lower T4 to upper T5 levels. Red arrows indicate the extraluminal portion of the tumour extending beyond the tracheal wall. (A, E) Initial chest CT performed during a routine health check‐up in a 45‐year‐old woman revealed a 14 mm endotracheal nodule involving the right lateral wall of the upper trachea without prominent extraluminal component. (B, F) Two weeks after bronchoscopic debulking, the endobronchial portion had significantly decreased without progression of the extraluminal component. (C, G) At 20‐month follow‐up post‐debulking, no recurrence of the endotracheal lesion was observed. However, interval growth of the extraluminal component was noted. (D, H) Post‐steroid therapy (4 years), the extraluminal lesion showed a notable reduction in size.

A 45‐year‐old woman was referred to our clinic for evaluation of a tracheal mass measuring 17 mm in its longest axial dimension (Figure 1). She was a never‐smoker with no significant past medical history and was asymptomatic. The lesion was incidentally discovered during a routine health check‐up. Her complete blood count, comprehensive metabolic panel, coagulation profile, erythrocyte sedimentation rate, C‐reactive protein and lactate dehydrogenase were all within normal limits and unremarkable.

**FIGURE 2:**
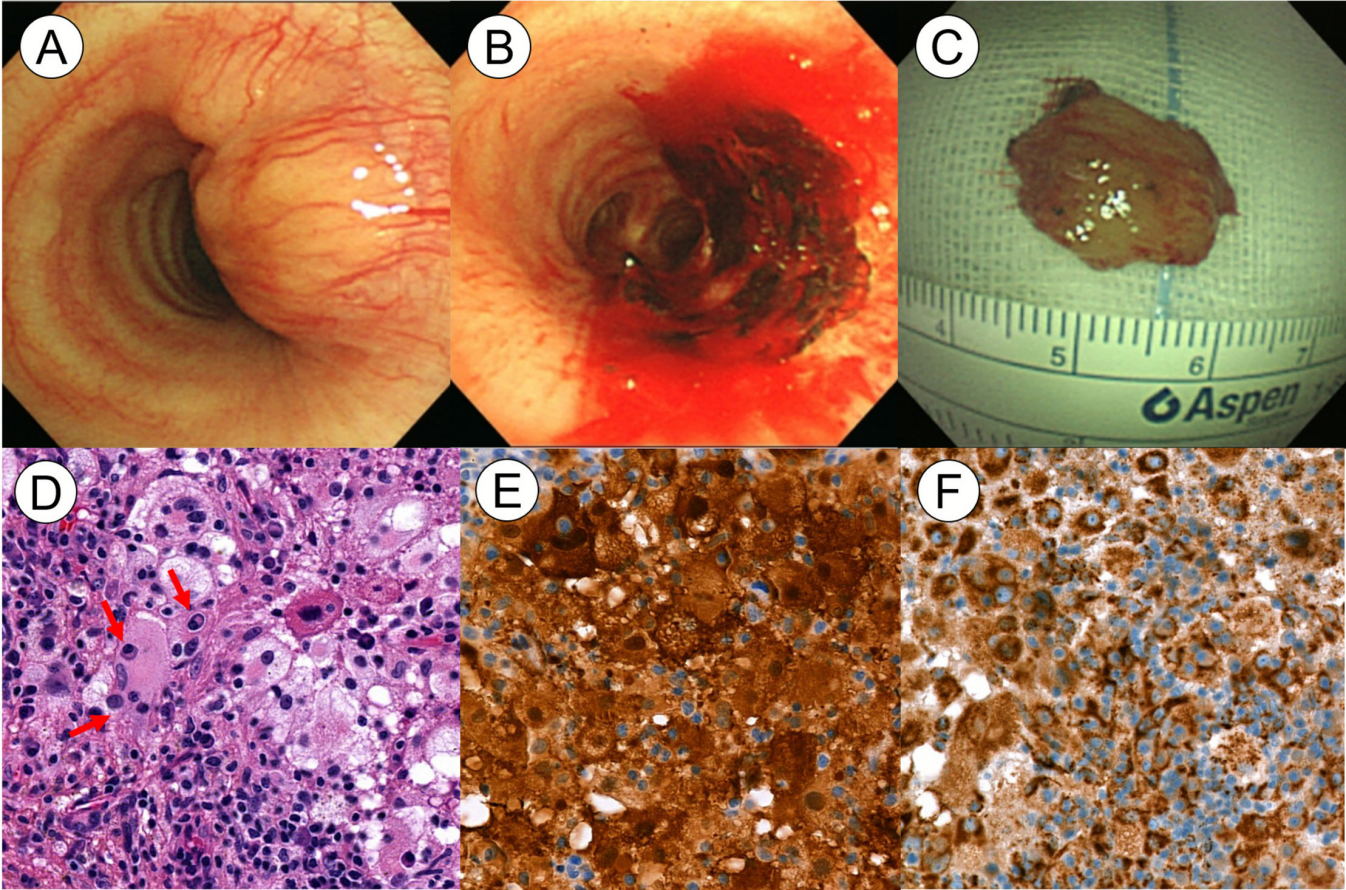
Bronchoscopic images and histopathological features. (A) Initial bronchoscopic examination revealed a broad‐based, protruding nodule causing approximately 50% luminal narrowing. (B) Following mechanical resection of the tumour via rigid bronchoscopy, the lesion base was cauterised using a diode laser. (C) The resected gross specimen measured approximately 15 mm in greatest dimension. (D) Haematoxylin and eosin staining reveals numerous large histiocytes with abundant pale cytoplasm. Emperipolesis (engulfment of intact lymphocytes) is observed and indicated by red arrows. (E) Immunohistochemical staining shows diffuse positivity for S100 in histiocytes. (F) Histiocytes also exhibit strong cytoplasmic positivity for CD68. All histopathological images are shown at ×200 magnification.

The mass was removed bronchoscopically using rigid bronchoscopy for both diagnostic and therapeutic purposes (Figure 2). It appeared as a broad‐based nodule protruding from the tracheal wall, causing approximately 50% luminal obstruction. The protruding portion, measuring approximately 15 mm, was mechanically resected, followed by diode laser cauterization of the base.

After multidisciplinary discussions involving pathologists, radiologists, oncologists, and pulmonologists, the lesion was diagnosed as RDD. Immunohistochemical staining showed positivity for S100 and CD68 in histiocytes (Figure 2). Haematoxylin–eosin staining revealed abundant large histiocytes throughout the specimen. Characteristic ‘Rosai–Dorfman cells’—large polygonal histiocytes positive for S100 and demonstrating emperipolesis—are highlighted in Figure 2D (indicated by red arrows).

Systemic evaluation through history taking and physical examination revealed no evidence of extrapulmonary involvement. For staging purposes, F‐18 fluorodeoxyglucose positron emission tomography/computed tomography was performed and showed no abnormal hypermetabolic lesions apart from the known tracheal mass. Autoimmune diseases and malignancies potentially associated with RDD were ruled out. Serum immunoglobulin levels were also within normal limits.

Given the absence of symptoms, the residual extratracheal lesion was initially managed conservatively without treatment. However, follow‐up chest computed tomography 20 months after resection demonstrated an increase in the size of the extratracheal component. Consequently, oral corticosteroid therapy was initiated, starting with 30 mg of prednisolone daily for 2 months (Figure 1), followed by gradual tapering over the next 7 months. The lesion subsequently decreased in size and has remained stable for 4 years following the completion of steroid therapy.

## 3 Discussion

RDD, also known as sinus histiocytosis with massive lymphadenopathy, is a rare non‐Langerhans cell histiocytosis characterised by the proliferation of distinctive histiocytes within lymph nodes and extranodal tissues. First described by Rosai and Dorfman in 1969, RDD typically presents with painless bilateral cervical lymphadenopathy. However, extranodal involvement occurs in approximately 43% of cases and may affect sites such as the skin, central nervous system and bones [2, 3].

The aetiology of RDD remains unclear. Some cases have been linked to viral infections, including Epstein–Barr virus, cytomegalovirus, human herpesviruses, and human immunodeficiency virus, as well as somatic mutations in genes such as NRAS, KRAS, MAP2K1 and ARAF [4, 5, 6, 7]. RDD may also coexist with hematologic malignancies such as lymphoma, myelodysplastic syndrome, leukaemia or other neoplasms including sarcoma [8, 9, 10, 11]. Autoimmune diseases are present in approximately 10% of RDD patients, and some cases exhibit increased immunoglobulin G4‐positive (IgG4^+^) plasma cells, warranting evaluation of the IgG4/IgG ratio [12, 13].

Histologically, RDD is characterised by large histiocytes with abundant pale cytoplasm, a hypochromatic nucleus, and a prominent nucleolus. Emperipolesis—the presence of intact lymphocytes or plasma cells within histiocyte cytoplasm—is a supportive but not mandatory diagnostic feature. Immunohistochemical staining typically shows positivity for S100, CD68 and CD163, while CD1a is negative [14]. Although CD1a staining could not be performed because of the limited amount of residual tissue in our case, the combination of histopathologic and immunohistochemical findings (S‐100 and CD68 positivity with cytokeratin negativity) supported the diagnosis of Rosai–Dorfman disease.

While classical RDD involves massive bilateral cervical lymphadenopathy, systemic symptoms such as fever, weight loss and night sweats may also be present. Extranodal disease is not uncommon, occurring in more than 40% of patients, with frequent involvement of the skin, nasal cavity, bone and orbit [14]. According to the consensus multidisciplinary recommendations proposed by the American Society of Haematology, “*No uniform approach has been delineated for RDD*, *and treatment is best tailored to the individual clinical circumstances* [2].” Various therapeutic options, ranging from simple observation to surgical resection, corticosteroids, chemotherapy, and radiotherapy, have been described. In a review of 12 reported cases of tracheobronchial RDD, diverse treatment modalities were employed, including observation (*n* = 4), bronchoscopic treatment (*n* = 5), and surgical resection (*n* = 2), as well as systemic therapies such as corticosteroids, chlorambucil, and methotrexate [15].

In this report, we describe an exceptionally rare case of RDD confined solely to the trachea. To the best of our knowledge, only a few cases of tracheal involvement have been previously reported [2]. Given the lack of standardised treatment guidelines, our case may offer insights into the management of airway‐limited RDD. The patient underwent endobronchial resection via rigid bronchoscopy followed by systemic corticosteroid therapy for the residual extratracheal lesion. This approach resulted in disease stabilisation for 4 years after therapy completion.

RDD is a heterogeneous disease that may occur in isolation or in association with autoimmune or neoplastic conditions. While observation may suffice in many cases, treatment should be individualised based on the extent and severity of the disease. For rare cases involving the airway, a combination of local debulking and corticosteroids may be an effective therapeutic option. This case highlights the importance of considering uncommon diseases such as RDD in the differential diagnosis of tracheal masses and demonstrates that localised disease can be successfully managed with a conservative, multidisciplinary approach.

## Author Contributions

T.Y. participated in the study design, data collection and drafting of the manuscript. J.H. and B.L. contributed to data collection and interpretation, and revised the manuscript. B.‐H.J. participated in the study design and data collection, and also revised the manuscript. All authors read and approved the final version of the manuscript.

## Funding

The authors have nothing to report.

## Ethics Statement

This study was approved by the Institutional Review Board (IRB) of Samsung Medical Center (Approval No. 2025‐04‐040‐001).

## Consent

The authors declare that written informed consent was obtained for the publication of this manuscript and accompanying images and attest that the form used to obtain consent from the patient complies with the journal requirements as outlined in the author guidelines.

## Conflicts of Interest

The authors declare no conflicts of interest.

## Data Availability

The data that support the findings of this study are available on request from the corresponding author. The data are not publicly available due to privacy or ethical restrictions.
